# Mandibular shape prediction using cephalometric analysis: applications in craniofacial analysis, forensic anthropology and archaeological reconstruction

**DOI:** 10.1186/s40902-020-00282-3

**Published:** 2020-10-31

**Authors:** Ahmed Omran, David Wertheim, Kathryn Smith, Ching Yiu Jessica Liu, Farhad B. Naini

**Affiliations:** 1 Kingston Hospital NHS Foundation Trust, Galsworthy Road, Kingston upon Thames, KT2 7QB UK; 2 School of Computer Science and Mathematics, Faculty of Science, Engineering and Computing, Kingston University, Penrhyn Road, Kingston upon Thames, Surrey KT1 2EE UK; 3 Face Lab, Liverpool John Moores University, Liverpool Science Park IC1 131 Mount Pleasant, Liverpool, L3 5TF UK; 4 Kingston Hospital NHS Foundation Trust, Galsworthy Road, Kingston upon Thames, KT2 7QB UK; 5 Maxillofacial Unit, St George’s Hospital & Medical School, Blackshaw Road, London, SW17 0QT UK

**Keywords:** Mandible, Shape, Cephalometric analysis, Orthognathic surgery, Forensic anthropology

## Abstract

**Background:**

The human mandible is variable in shape, size and position and any deviation from normal can affect the facial appearance and dental occlusion.

**Objectives:**

The objectives of this study were to determine whether the Sassouni cephalometric analysis could help predict two-dimensional mandibular shape in humans using cephalometric planes and landmarks.

**Materials and methods:**

A retrospective computerised analysis of 100 lateral cephalometric radiographs taken at Kingston Hospital Orthodontic Department was carried out.

**Results:**

Results showed that the Euclidean straight-line mean difference between the estimated position of gonion and traced position of gonion was 7.89 mm and the Euclidean straight-line mean difference between the estimated position of pogonion and the traced position of pogonion was 11.15 mm. The length of the anterior cranial base as measured by sella-nasion was positively correlated with the length of the mandibular body gonion-menton, *r* = 0.381 and regression analysis showed the length of the anterior cranial base sella-nasion could be predictive of the length of the mandibular body gonion-menton by the equation 22.65 + 0.5426*x*, where *x* = length of the anterior cranial base (SN). There was a significant association with convex shaped palates and oblique shaped mandibles, *p* = 0.0004.

**Conclusions:**

The method described in this study can be used to help estimate the position of cephalometric points gonion and pogonion and thereby sagittal mandibular length. This method is more accurate in skeletal class I cases and therefore has potential applications in craniofacial anthropology and the ‘missing mandible’ problem in forensic and archaeological reconstruction.

## Background

The mandible forms the lower jaw in the human face and contains the mandibular dentition. The mandible lies beneath the maxilla and articulates with the temporal part of the temporal bone of the skull at the glenoid fossae bilaterally at the temporomandibular joint (TMJ).

The shape, size and the relationship of the mandible to the maxilla and cranial base is of primary interest as it can influence the position and appearance of the facial soft tissues as viewed in profile. As the mandibular dentition is contained in the alveolar process of the mandible, any deviation in the shape, size or relationship of the mandible to the maxilla may be an aetiological factor in a presenting malocclusion [1]. Many factors have been implicated in influencing mandibular shape, and it has been suggested that existing cranial form is associated with the shape of the mandible [2].

In orthodontics and craniofacial reconstructive surgery, the shape, size and relationship of the mandible to the maxilla and cranial base is first assessed clinically and this may be supplemented by requesting a lateral cephalometric radiograph (LCR) in the natural head position [3].

LCRs are usually traced, either by hand or by digital software to provide a wide range of both linear and angular measurements dependent on the points plotted and the analyses used. These are then compared to a set of normal values based on an existing accepted dataset for an ethnic group so that it is possible to quantify any deviations in the examined individual relative to the referenced normal population. A traced LCR can provide a large amount of data, and therefore a plethora of analyses have been described to aid orthodontic diagnoses and treatment planning.

Of these analyses, the Sassouni analysis [4] is based on a study by Viken Sassouni published in 1955 in the American Journal of Orthodontics. Sassouni suggested that disturbances or disproportions in facial architecture may be an important aetiological feature of malocclusion in orthodontics. His arcial analysis involved extending several horizontal reference planes posteriorly which, in a well-proportioned face, should all converge at the same point, the origin, known as point O.

He described the following planes: mandibular base plane, OG—a plane tangent to the inferior border of the mandible; occlusal plane, OP—a plane through the mesial cusps of the permanent upper and lower first molars and the incisal edges of the upper and lower central incisors; palatal plane, ON—a plane perpendicular to the midsagittal plane, going through ANS-PNS; and the anterior cranial base or basal plane, OS’—a plane parallel to the axis of the upper contour of the anterior cranial base and tangent to the inferior border of the sella turcica (ST). In a well-proportioned face, all four of these planes should converge at point O, which would usually be situated just posterior to the occipital region.

He also described two arcs: anterior arc—the arc of a circle, between the anterior cranial base plane and mandibular plane (MdP) with O as a centre and O-anterior nasal spine (ANS) as the radius which should pass through pogonion (Pog), the incisal edge of the upper central incisor, ANS, nasion and fronto-ethmoid junction and the posterior arc—the arc of a circle, between OS’ and OG, with O as a centre and OSp as the radius which passes through gonion (Go) and Sp (Sp is the most posterior point on the rear margin of the ST).

In addition to this, he described three shapes of the palate and three shapes of the mandible and noticed associations between the two. With a horizontal palate, he found that the mandible is often curved; with a convex palate, he found that the mandible is often oblique and with a concave palate, he found that the mandible is often horizontal.

Therefore, using parts of this analysis, it may be possible to estimate the position of two points on the mandible, both Go and Pog by using Sassouni’s reference planes combined with the posterior arc and anterior arc Sassouni originally described.

In this study, an adapted version of the Sassouni analysis was used to try and estimate the positions of cephalometric points Go and Pog. The length of the anterior cranial base as measured by sella-nasion (SN) and the length of the body of the mandible as measured by gonion-menton (Go-Me) were studied to see if there was a relationship between the two. The shape of the palate and the shape of the mandible were also studied to assess whether there were any correlations between the shape of the palate and the shape of the mandible.

## Materials and methods

A retrospective observational exploratory study design was used, requiring only LCRs which had already been taken of patients for orthodontic assessment or diagnostic purposes. Ethical approval was sought and given by the Health Research Authority (Project ID 245045) for carrying out this study.

A sample size of 100 LCRs was decided by joint consultation with a statistician. A previous literature review on studies which assessed the shape of the mandible by cephalometry was done by the author, AO. Previous analogous studies showed a range of sample sizes between 41-141 subjects; these sample sizes were used to inform the choice of sample size as a power calculation would be limited as this type of study had not been carried out before.

The sample size of 100 was selected by the clinical care team from Kingston Hospital National Health Service Foundation Trust records on Picture Archiving and Communication System. A filter was placed on LCRs taken from 01/01/2014-01/01/2018 and the clinical care team consecutively looked at each LCR, applying the inclusion and exclusion criteria until the sample size of 100 was reached.

Inclusion criteria:

- Has had a grade one (excellent) or grade two (diagnostically acceptable) LCR taken preoperatively or pre-orthodontic treatment

Exclusion criteria:

- Has a grade three (diagnostically unacceptable) LCR
- Has a diagnosed craniofacial deformity (e.g. cleft lip and palate, acromegaly)
- Post-orthognathic surgery or orthodontic fixed or removable appliance in situ
- History of surgical intervention for trauma to the mandible (plates/pins visible)
- Local diagnosed pathology affecting the craniofacial skeleton or cranial base

### Sample demographics

Of the 100 lateral cephalometric radiographs, 56 were female and 44 were male. The average age was 21.74 years with a minimum of 13 years and a maximum of 48 years. There were a mix of ethnicities included, with 44 White Caucasian, 27 classified as Other Ethnic Group, 15 Unspecified, 6 Asian, 5 Black African and 3 Mixed. In terms of the skeletal patterns, 28 were class I, 26 were skeletal class II and 46 were skeletal class III. In terms of the incisor relationships, 34 were class I, 14 were class II division 1, 5 were class II division 2 and 47 were class III.

Each subject was pseudonymised and linked to their respective LCR and these were uploaded onto Dolphin Imaging Plus™ (Ver 11.95 SP2) and passed to the research team for digital cephalometric tracing, application of the Sassouni analysis and data collection.

Each LCR was traced digitally using Dolphin Imaging Plus™ (Ver 11.95 SP2) using the Sassouni+ analysis by the author, AO. Each cephalometric point was plotted visually using standard landmark definitions. For bilateral landmarks that were not superimposed, an average of the two was plotted. Magnification calibration was done for each individual LCR by plotting a known distance of 10 mm on the LCR by using the marking ruler present on each LCR. Both linear and angular measurements up to 0.1 mm and 0.1° accuracy were taken.

**Fig. 1:**
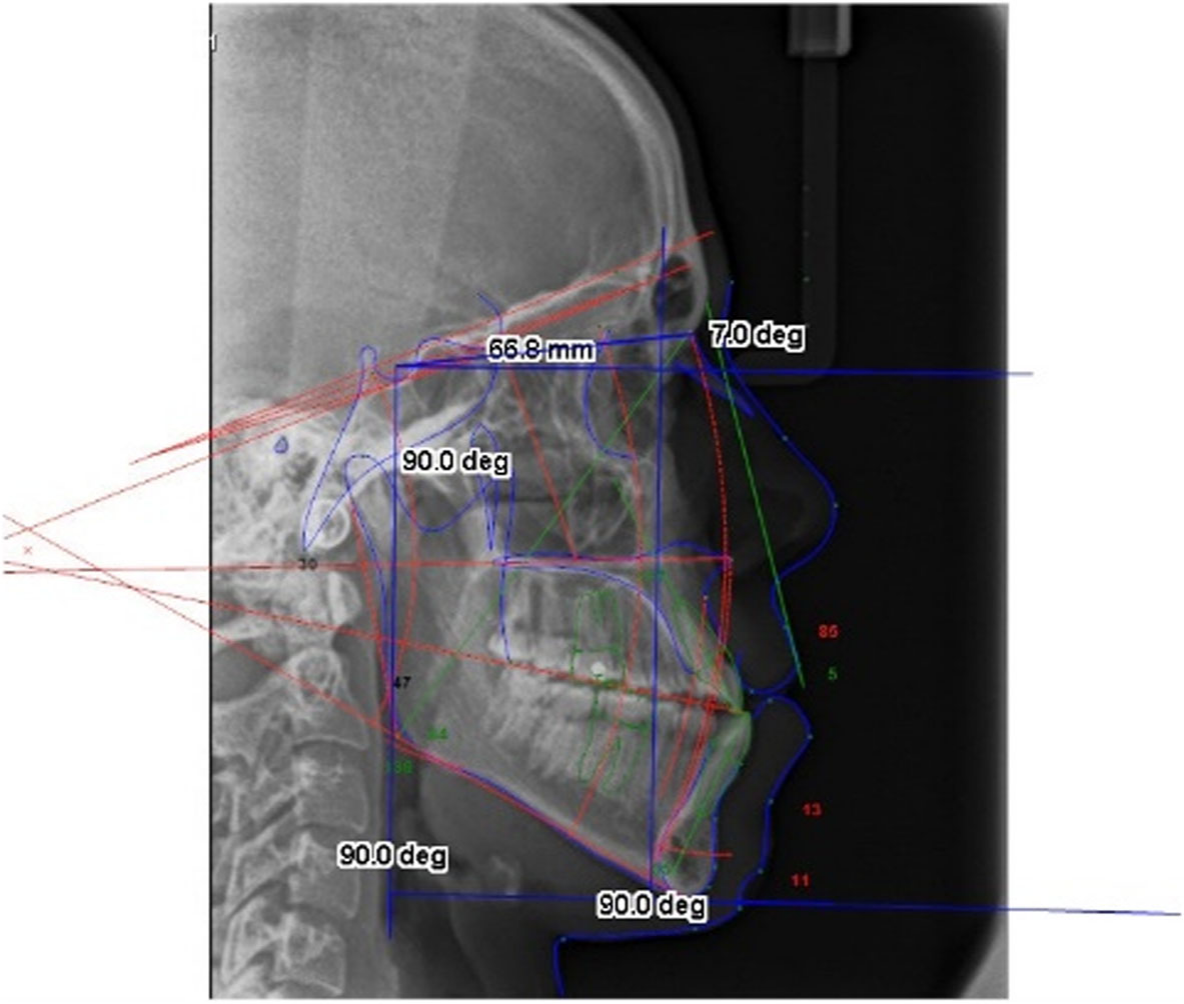
Image on Dolphin software depicting the posterior arc and anterior arc intersection with the mandibular plane

The points Go (*x*,*y*) and Pog (*x*,*y*) as well as a number of other cephalometric landmarks were traced on the first digital tracing, prior to Sassouni+ analysis. Sassouni+ analysis was then applied onto a duplicate copied second tracing. After Sassouni+ analysis, the estimated position of Go (*x*,*y*) was found using the intersection between the posterior arc and the MdP as determined by Go-Me. The estimated position of Pog (*x*,*y*) was found using the intersection between the anterior arc and the MdP (Fig. 1).

The Cartesian co-ordinates (*x*,*y*) for Go and Pog were calculated by using SN line and a ‘true’ horizontal reference line 7.0° clockwise from the SN line. Point sella (S) was used as the Cartesian co-ordinate graph origin (0,0) and a 90.0-degree perpendicular line to the ‘true’ horizontal reference line was dropped vertically from S to form a ‘true’ vertical reference line. Two more 90.0° perpendicular lines were then drawn from both the ‘true’ horizontal and vertical reference lines to form standardised *x-* and *y*-axis upon which the Cartesian co-ordinates (*x*,*y*) could be measured and standardised between LCRs (Fig. 1). Distances were calculated in millimetres and calibrated individually for each LCR.

Prior to data collection, a random LCR was selected and traced digitally and the Sassouni analysis was carried out as described previously. This was checked for agreement with the Chief Investigator (FBN) to ensure validity.

AO then used an online computerised random number generator to randomly select 12 LCRs for tracing, and then applied Sassouni’s+ analysis and collected the data. The same data sets were collected for the same 12 LCRs two weeks after the initial measurement by the same examiner AO, to assess intra-examiner agreement and repeatability.

All data for the entire sample were then collected by a single examiner, AO, and inputted in Microsoft Excel™ (Version 1909). The collected data were then statistically analysed by DW using Minitab v19 (Minitab LLC., USA) and SPSS v26 (IBM Corp., USA). Data were tested for consistency with a normal distribution using the Ryan-Joiner test in Minitab and accordingly analysed using parametric or non-parametric tests; *p* < 0.05 was considered to indicate statistical significance.

## Results

To assess intra-examiner agreement, two measurements were taken for the estimated and traced (*x*,*y*) co-ordinates for Go and Pog on two separate occasions done two weeks apart under the same conditions by the same examiner, AO.

The Bland-Altman method [5] and the intraclass correlation coefficient (ICC) with a two-way fixed mixed-effects model were used to assess intra-examiner agreement [6] (Table 1).

**Table 1:** Mean differences and average measure intraclass correlation coefficients between 12 LCR measurements taken two weeks apart

	Estimated Go(x) diff	Estimated Go(y) diff	Traced Go(x) diff	Traced Go(y) diff	Estimated Pog(x) diff	Estimated Pog(y) diff	Traced Pog(x) diff	Traced Pog(y) diff
Mean	0.10	0.23	− 0.20	0.00	2.55	2.75	− 0.44	− 1.16
sd	0.49	0.55	0.78	0.38	5.24	3.42	0.92	2.05
2sd	0.99	1.10	1.57	0.76	10.48	6.85	1.85	4.11
ICC	0.998	0.997	0.998	0.999	0.786^a^	0.960	0.996	0.990

^a^Significant. *SD* Standard deviation, *ICC* Intraclass correlation coefficient

The results show excellent repeatability for all measurements (ICC > 0.900) except for the Estimated Position of Pog (*x*) which only had good repeatability ICC = 0.786 (0.305–0.937) 95% CI and mean difference 2.55, SD = 5.24.

**Fig. 2:**
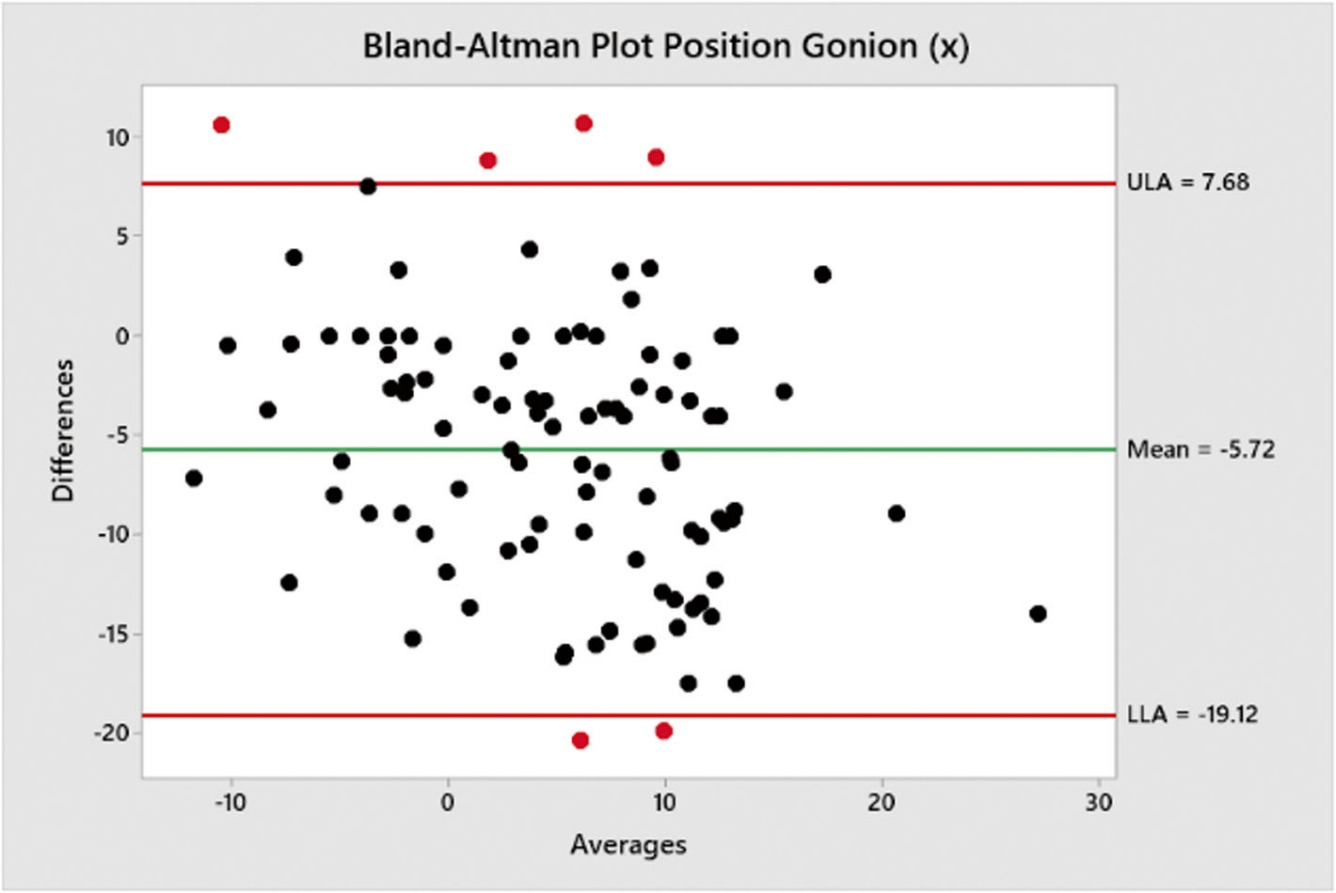
Bland-Altman plot showing mean difference between traced position of Go (*x*) co-ordinate and estimated position by Sassouni+ analysis versus mean (average); the mean difference and limits of agreement are shown

**Fig. 3:**
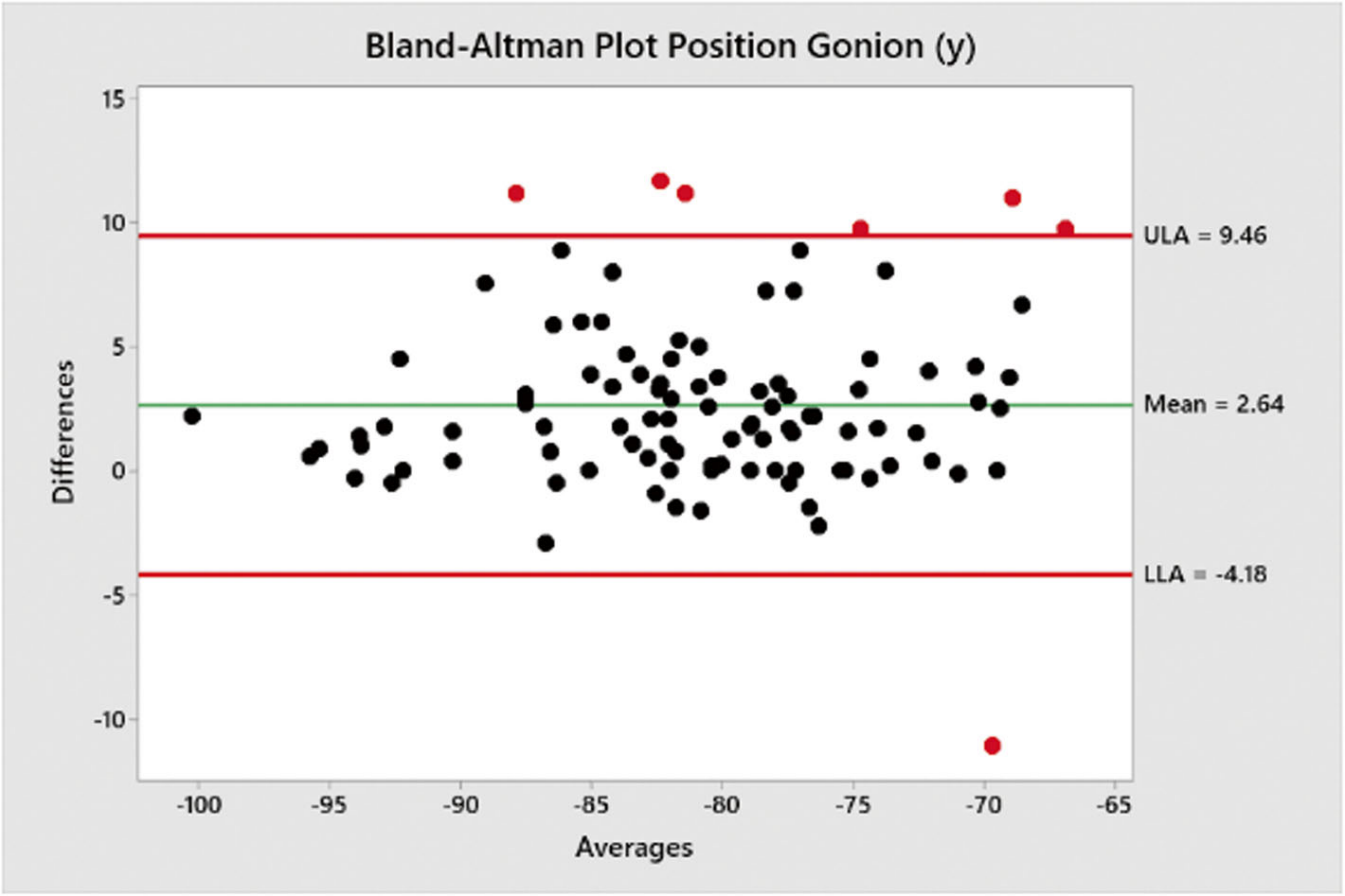
Bland-Altman plot showing mean differences between traced position of Go (*y*) co-ordinate and estimated position by Sassouni+ analysis versus mean (average); the mean difference and limits of agreement are shown

**Fig. 4:**
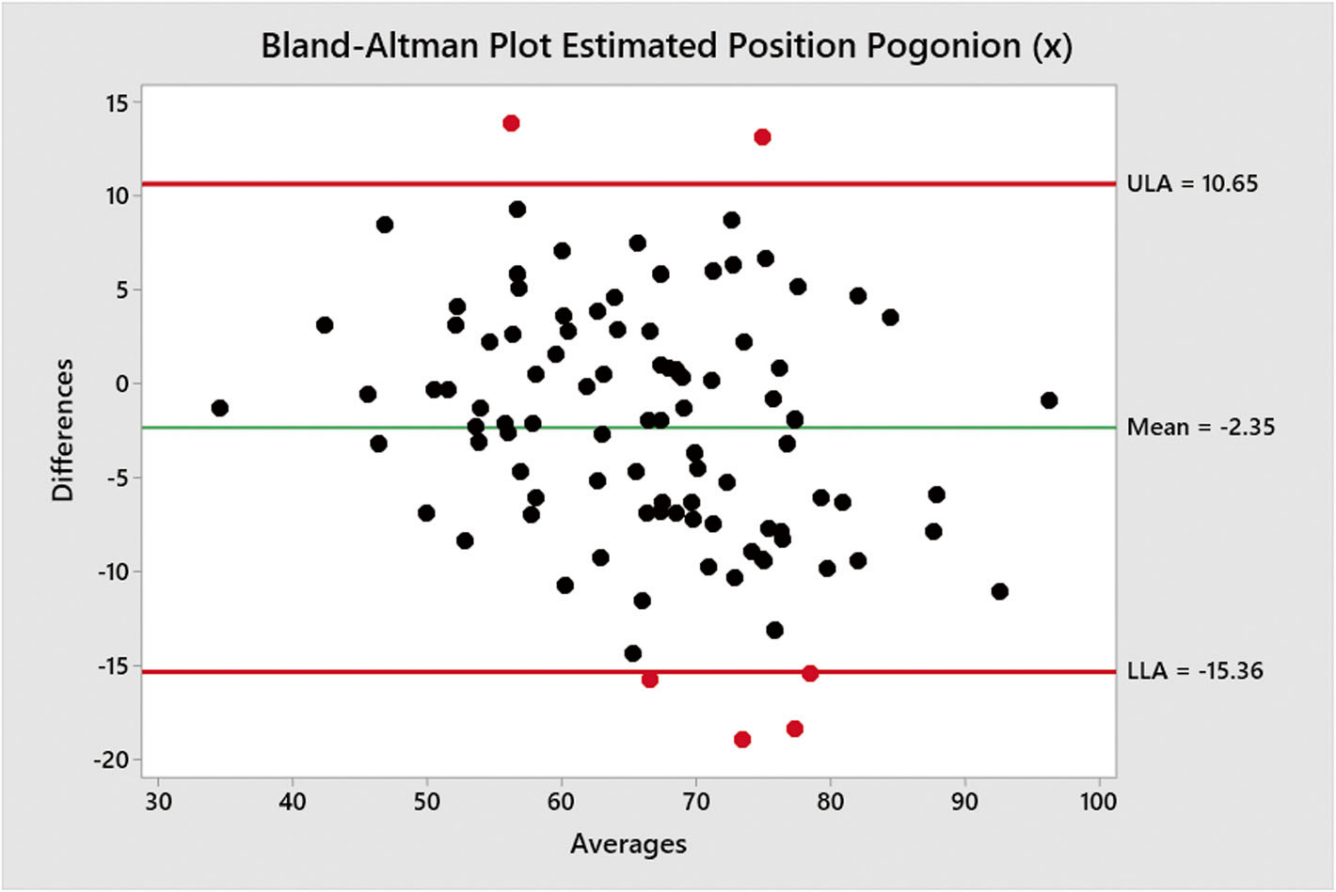
Bland-Altman plot showing mean differences between traced position of Pog (*x*) co-ordinate and estimated position by Sassouni+ analysis versus mean (average); the mean difference and limits of agreement are shown

**Fig. 5:**
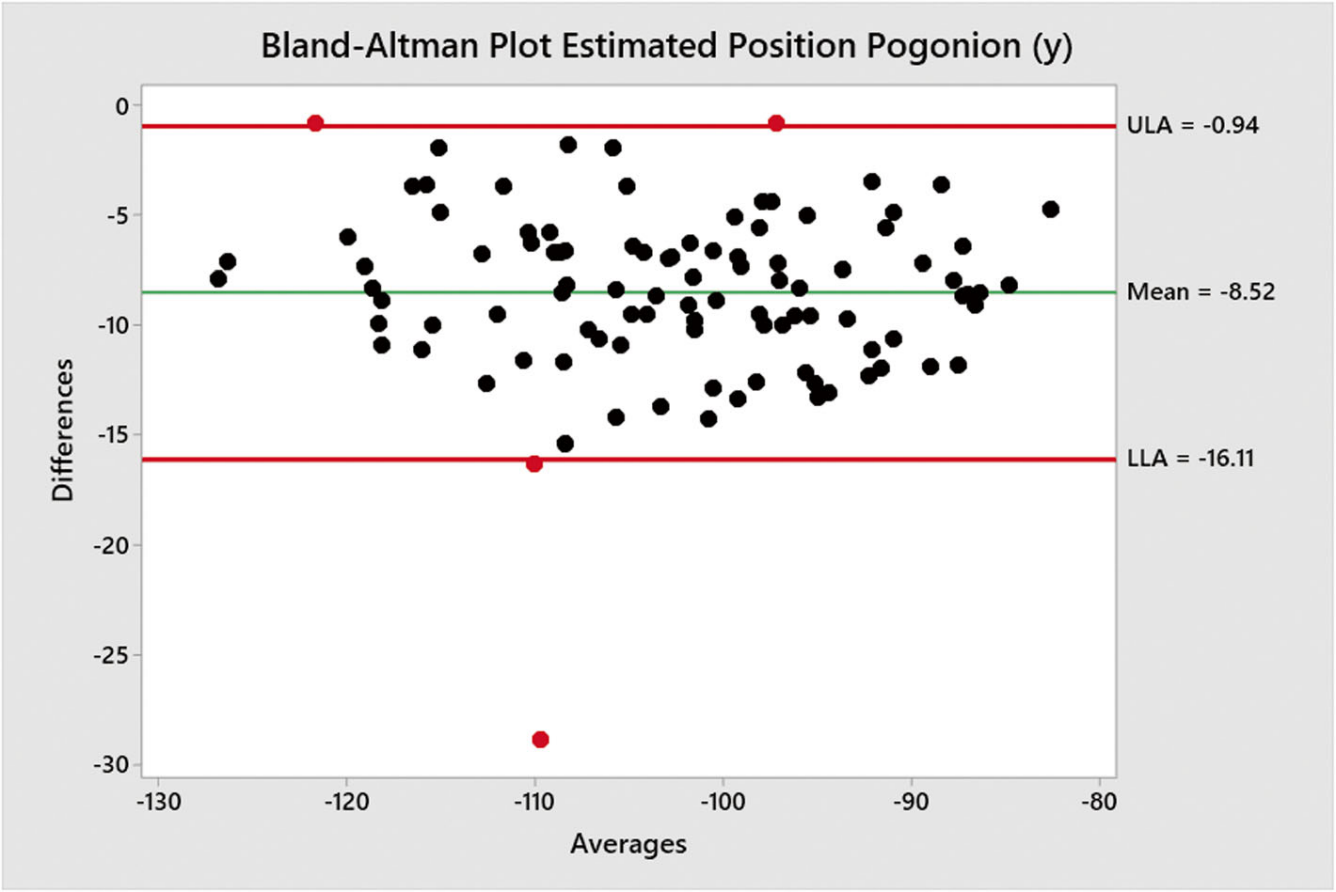
Bland-Altman plot (estimated–traced of negative measured positions) between traced position of Pog (*y*) co-ordinate and estimated position by Sassouni+ analysis versus mean (average); the mean difference and limits of agreement are shown. There appears to be one outlier with a mean value of − 109.8 and for which there was a difference of − 28.9

Bland-Altman plots performed using a macro in Minitab v19 were used to measure mean differences and show upper and lower limits of agreements between the traced positions of Go (Figs. 2 and 3) and Pog (Figs. 3 and 4) when compared to the positions estimated by Sassouni+ analysis (Fig. 5).

The mean difference between the traced position and estimated position of Go (*x*) co-ordinate was − 5.72 mm (− 19.12–7.68).

The mean difference between the traced position and estimated position of Go (*y*) co-ordinate was − 2.64 mm (− 4.18–9.64).

The mean difference between the traced position and estimated position Pog (*x*) co-ordinate was − 2.35 mm (− 15.36–10.65).

The mean difference between the traced position and estimated position Pog (*y*) co-ordinate was − 8.52 mm (− 16.11 to − 0.94).

**Fig. 6:**
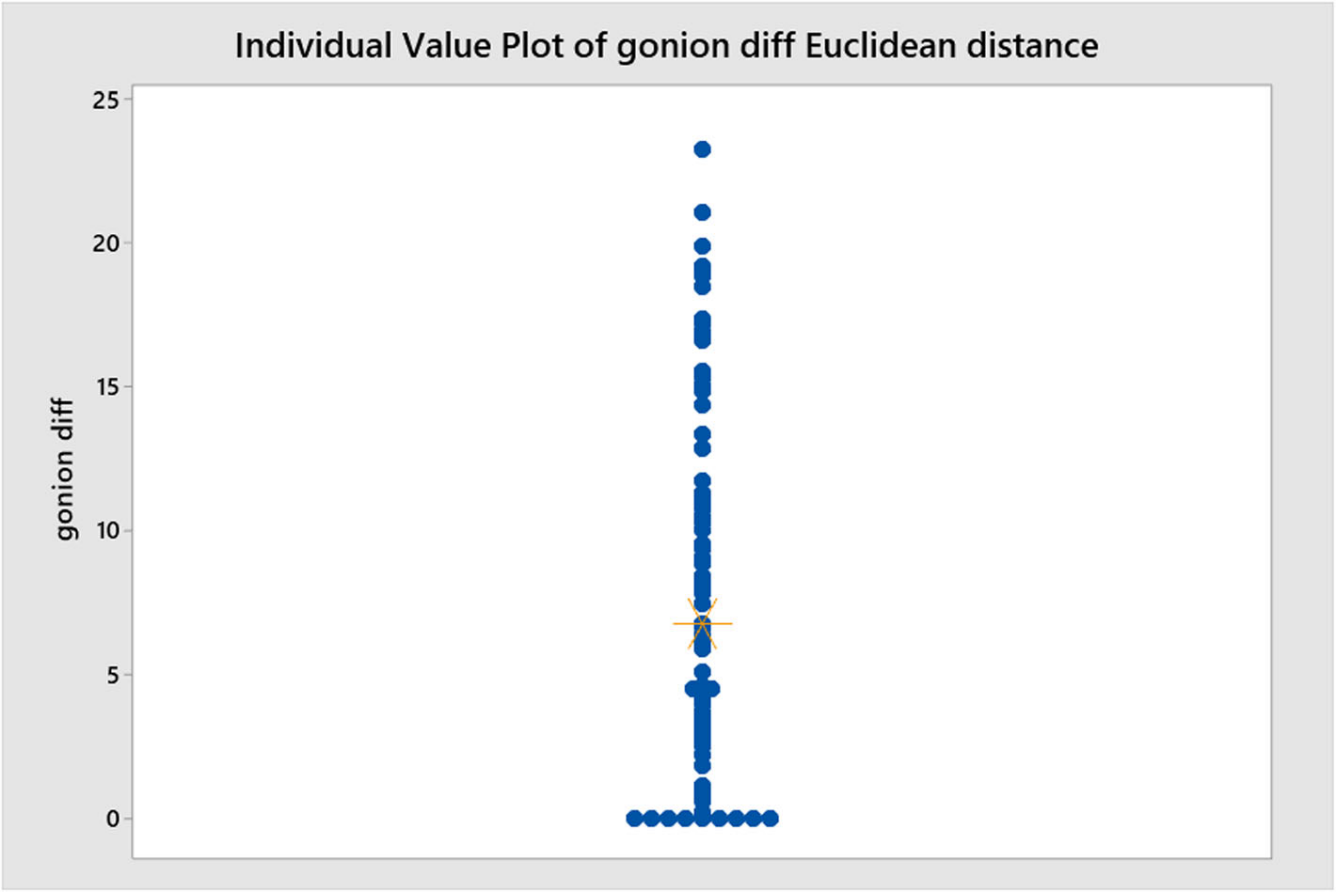
Individual value plot showing mean distances between traced position of Go and estimated position by Sassouni+ analysis

**Fig. 7:**
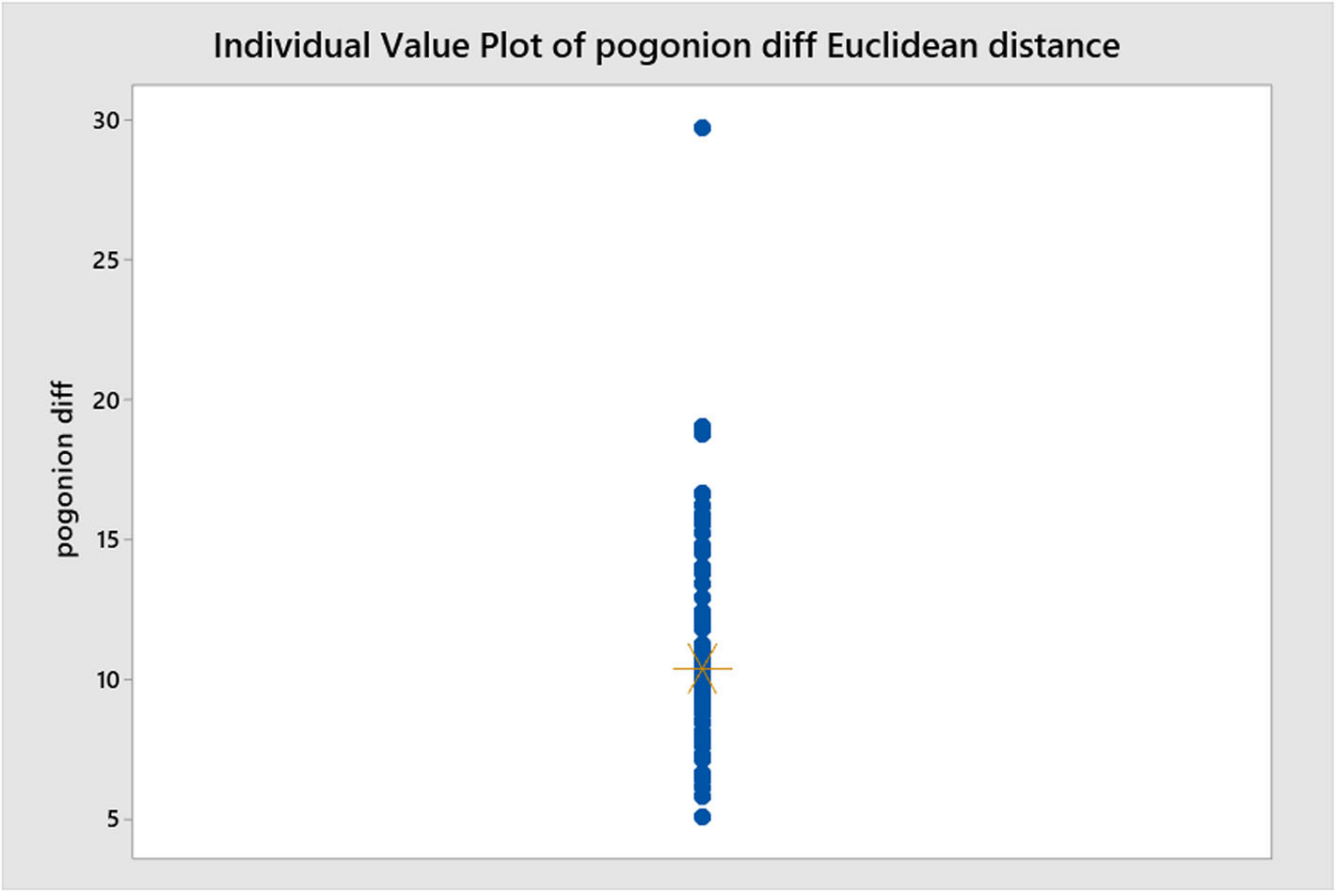
Individual value plot showing mean distances between traced position of Go and estimated position by Sassouni+ analysis

Euclidean straight-line distance values were calculated for both Go (Fig. 6) and Pog (Fig. 7) using both (*x*) and (*y*) co-ordinates to give a single value. The Ryan-Joiner test in Minitab was used to assess normality and it was found that the data followed a non-normal distribution.

The mean straight-line difference between traced position and estimated position Go was 7.89 mm (3.12–11.24) interquartile range (IQR).

The mean straight-line difference between the traced position and estimated position Pog was 11.15 mm (9.06–12.38) (IQR).

**Fig. 8:**
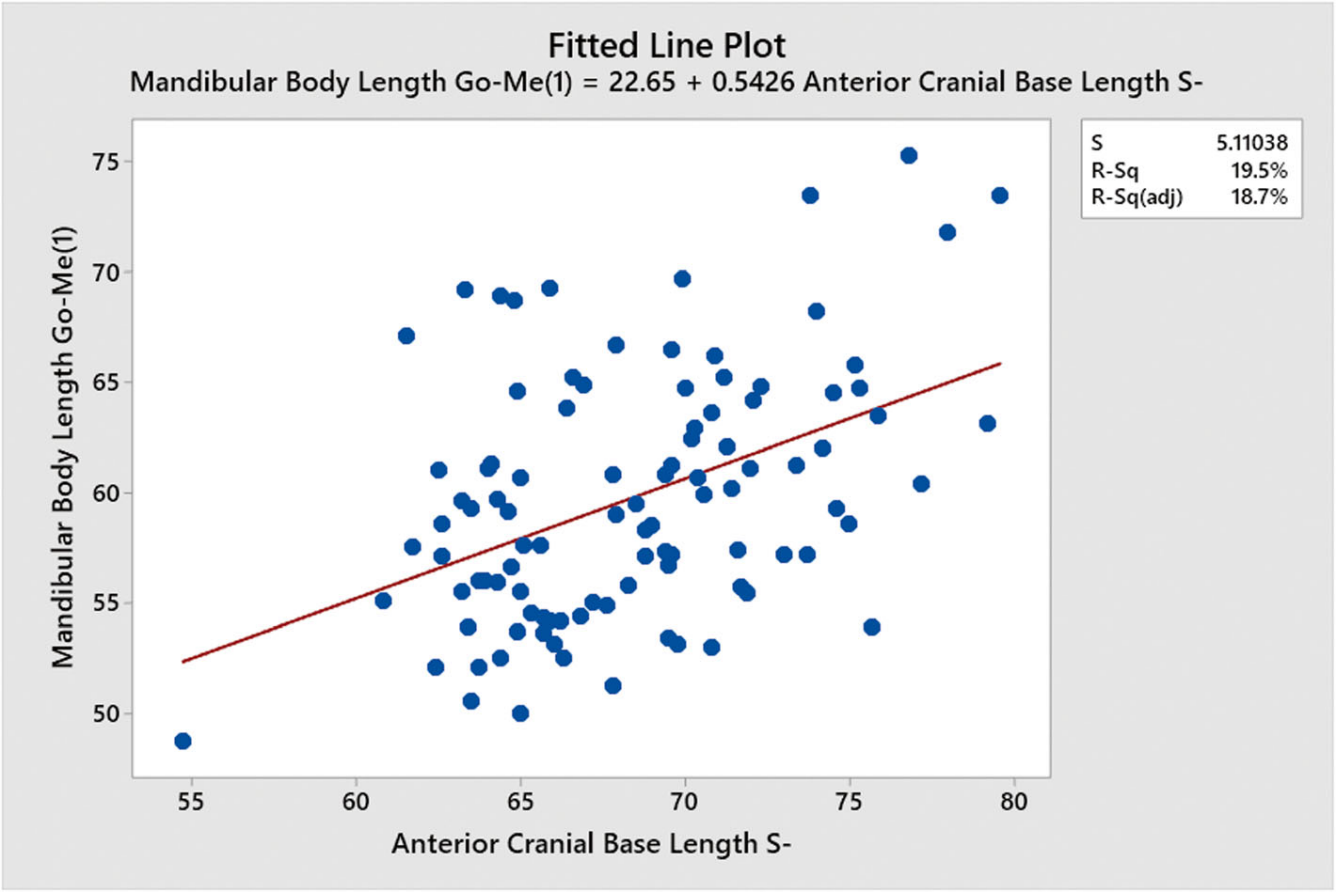
Regression analysis between the length of the anterior cranial base (SN) and the length of the mandible (Go-Me)

Pairwise Spearman’s correlation between the length of the anterior cranial base (SN) and the length of the mandibular body (Go-Me) was calculated as *r* = 0.381 (0.193–0.542) 95%CI (*p* < 0.001). Regression analysis between of length of the anterior cranial base (SN) and the length of the mandible (Go-Me) is shown in Fig. 8.

The predictive equation for the length of the mandibular body (Go-Me) was 22.65 + 0.5426*x*, where *x* = length of the anterior cranial base (SN) in millimetres.

To assess for an association between the shape of the palate and the shape of the mandible, a chi-squared test was carried out and is shown in Table 2.

**Table 2:** Association between the shape of the palate and the shape of the mandible

		Shape of palate			
		Concave	Convex	Horizontal	All
Shape of mandible	Curved	9	5	21	35
	Expected	9.800	8.050	17.150	
	Chi-square	0.0653	1.1556	0.8643	
	Horizontal	13	3	15	31
	Expected	8.680	7.130	15.190	
	Chi-square	2.1500	2.3923	0.0024	
	Oblique	6	15	13	34
	Expected	9.520	7.820	16.660	
	Chi-square	1.3015	6.5924^a^	0.08041	
	All	28	23	49	100
		Chi-square	DF	*p* value	
		15.328	4	0.004	

^a^ Significant. *DF* Degrees of freedom

The results show that there is a significant association between the shape of the palate and the shape of the mandible, *p* = 0.004. In order to further assess the apparent association of convex palate with oblique mandible, whether the palate was convex was compared with whether the mandible was oblique. Because there are nine different combinations from the original data, the Bonferroni correction was used. Using Fisher’s exact test, *p* = 0.0008 when comparing whether the palate was convex with whether the mandible was oblique. There were no other significant associations between the shape of the palate and the shape of the mandible when taking into account the multiple comparisons correction.

## Discussion

Of all 100 LCRs analysed in this study, not one had all four of Sassouni’s described planes converge precisely at point O. This contrasts with Sassouni’s original study where 16 of his sample had all four planes converge at point O. This result may be explained by a difference in the samples selected between Sassouni’s study and this study. Sassouni’s original sample were 7–15-year-old Mediterranean Caucasians. The sample used in this study were aged between 13 – 48 years of age and of any ethnicity. Therefore, point O which is required as a centre of which to draw either the posterior arc or the anterior arc was a theoretical point used in this study, calculated automatically by Dolphin Imaging Plus™ Software by using an averaged angle of the four marked horizontal planes.

The results show that the mean difference for the estimated position of Go (*x*) was − 5.72 mm while Go (*y*) was − 2.64 mm relative to the traced position of Go (*x*,*y*). In context, these differences can be considered small, and therefore, the application of the Sassouni+ analysis is reasonably accurate in estimating the position of Go. The (*y*) co-ordinate estimation was more accurate than the (*x*) co-ordinate and this is to be expected as the intersection between the posterior arc and the MdP (Go-Me) was used to determine the estimated position of Go. As Go forms one of the posterior landmarks on the MdP, it is not surprising the vertical (*y*) co-ordinate was more accurate than the horizontal (*x*) co-ordinate.

The results also show that the mean difference for the estimated position of Pog (*x*) was − 2.35 mm while Pog (*y*) was − 8.52 mm. The difference in Pog (*x*) can be considered small; however, the difference in Pog (*y*) is relatively large; therefore, the application of the Sassouni+ analysis is reasonably accurate in estimating the horizontal position of Pog but inaccurate in estimating the vertical position of Pog. In contrast to the estimated position of Go, the (*y*) co-ordinate was far less accurate than the (*x*) co-ordinate, with the analysis consistently estimating the position of Pog (*y*) to be more inferior than the traced position of Pog (*y*) was. This is also to be expected, as the intersection between the anterior arc and the MdP (Go-Me) was used to determine the position of Pog. The traced position of Pog would always be expected to be above the MdP as it is not a landmark that is used in determining the MdP. Pog should always lie superior to menton (Me), which was one of the points used in determining the MdP in this study.

The Euclidean straight-line distance analyses show a 7.89-mm mean difference between the estimated position of Go and a 11.15-mm mean difference for Pog. The difference in accuracy between the two can be explained by the consistently inferior estimation of Pog (*y*) by the methodology of this study. Overall, it can be concluded that the application of Sassouni’s analysis can be reasonably accurate in determining the position of both Go and Pog when the position of the MdP (Go-Me) is already known.

There was a positive correlation coefficient of *r* = 0.381 between the anterior cranial base length (SN) and the length of the mandibular body (Go-Me). This result is also to be expected, as the length of the anterior cranial base increases, it would be expected that the length of the mandibular body (Go-Me) also increases to maintain facial proportion. Further regression analysis showed the length of the anterior cranial base (SN) could be used as a predictive variable for the length of the mandibular body using the equation 22.65 + 0.5426*x*, where *x* = length of the anterior cranial base (SN) in millimetres. This has application as a potential starting point in determining a predictive length of mandibular body (Go-Me) where the mandible is missing, however, caution has to be taken in solely using the cranial base as a predictor of mandibular length as it has been found that cranial base length correlated strongly with maxillary length but weakly with mandibular length [7].

The results also show that there is a significant correlation between convex-shaped palates and oblique-shaped mandibles. The oblique mandibular shape described by Sassouni shows typical features of a backward clockwise growth rotation pattern as described by Björk [8]. However, if the palate was concave or horizontal, then there was no association found with the shape of the mandible. This is in part likely due to the method of the study. With the method of the study, the author AO had to use ‘best fit’ by comparing the shape of the palate on the LCR with the diagrams described by Sassouni ([4], which introduced subjectivity in the assessment and potential bias. Furthermore, the concave and horizontal shapes of the palate as described by Sassouni were relatively similar to each other when compared with the convex shape of the palate, which had a much more characteristic shape that was readily identifiable. This meant that there was greater uncertainty when classifying a two-dimensional radiographic image of the palate on a LCR as either concave or horizontal.

Overall, the accuracy of the Sassouni analysis in estimating the positions of Go and Pog is surprisingly good when considering the sample selected. The sample consists of 100 LCRs taken in a secondary care orthodontic department and therefore an assumption could be made that most of the selected sample would likely have a greater degree of malocclusion with facial skeletal disproportion relative to the general population. This is because those who are considered to be in great need of orthodontic treatment [9] with more complex malocclusions requiring interdisciplinary care [10] are more likely to be referred to orthodontic secondary care in the UK. Bearing this in mind, the expectation would be that the Sassouni analysis would be inaccurate in estimating the positions of Go and Pog as most of the sample population would not be expected to have balanced facial proportions or a normal occlusion.

Interestingly, an exploratory finding is that in all 8 cases where the estimated Go (*x*) co-ordinates were exactly coincident with the traced Go (*x*) co-ordinates, every single case was identified as skeletal class I which suggests the analysis is most accurate in skeletal class I cases. This is an important consideration for future investigation.

The implications of these results are that there are some useful indicators on a LCR that can be used to help predict the two-dimensional shape of the mandible. The length of the anterior cranial base (SN) can be used to give an estimate of the length of the mandible at (Go-Me) using the given regression analysis equation 22.65 + 0.5426*x*, where *x* = length of the anterior cranial base (SN) in millimetres. Where the palate is a convex shape, it is likely that the shape of the mandible is oblique and that the individual is hyperdivergent. The Sassouni+ analysis can be used with a theoretical point O and posterior arcs and anterior arcs used to estimate the positions of Go and Pog respectively.

The validity of this approach can be questioned, considering the Sassouni analysis was originally designed for use when the reference planes converge exactly at the point of origin O. In this study, this did not occur in any of the selected sample, and therefore, a theoretical average O had to be used instead. In this way, the original analysis had to be adapted for use in this study and therefore the analysis ‘transformed’ the individual into one with average vertical proportions even if they were not. This likely explains why the adapted Sassouni’s analysis was more accurate in estimating the positions of Go and Pog than expected based on the study sample population characteristics.

A limitation of the method used to estimate the positions of Go and Pog in this study is that the method used the known MdP as determined by Go-Me as the inferior limit of intersection with the posterior and anterior arcs. Where the mandible may be missing, such as in forensic science or facial reconstruction, then a different inferior limit or plane of intersection with the posterior arc or the anterior arc will be required to estimate the *y* co-ordinates of both Go and Pog. This would be required before the Sassouni analysis is carried out as it forms one of the planes which may or may not converge with the other planes at point O. A method would have to be devised to extrapolate this, possibly by using the other angular measurements between the marked horizontal planes in order to determine the vertical position of the mandibular reference points. Orthlieb et al. [11] studied correlations between mandibular shape and lower facial height and found that the mandibular (gonial) angle (Articulare-go-chin point) had the strongest coefficient of correlation with lower facial height (*r* = 0.691) but with large dispersion, highlighting the difficulties with predicting vertical facial proportions from other measurements.

A further limitation of this study is that by design, this method is limited to two-dimensional shape prediction based on two-dimensional radiographic imaging, whereas the mandible is a complex three-dimensional object [12]. Transverse measurements such as bigonial width or any asymmetries [13] could not be assessed using this methodology. Three-dimensional imaging with further landmarks, measurements and volumetric data would be required in order to assess more complex differences in shape, size and position.

In this study, the focus was mainly on the Cartesian co-ordinates of landmarks Go and Pog. To more accurately predict the shape of the mandible, a greater number of cephalometric landmarks would be required in order to predict other parts of the mandible such as the mandibular ramus, condyle and coronoid process. A common shape analysis used in anthropology employs the use of geometrical morphometrics which uses Cartesian landmark co-ordinates that can capture shape variables. Linear measurements and angular measurements on their own are unreliable in predicting complex shapes as they combine shape and size together [14] and do not always include details of the more subtle aspects of the mandibular form [15].

A weakness of the Sassouni+ analysis and a number of other cephalometric analyses [16–18] is that they can be time consuming to carry out and like many cephalometric studies, are subject to error of the method with differences between examiners in landmark identification, and errors [19, 20] in both angular and linear measurements [21, 22]. This can affect the reliability and reproducibility of the data collected.

Predicting the shape of the mandible presents numerous difficulties as there can be significant differences in the shape, size and position of the mandible between individuals. Furthermore, the size and shape of the mandible is subject to changes with growth over time. Interestingly, between the ages of 16-99, Parr et al. [23] found that few mandibular measurements exhibited age-related changes, and most were affected by antemortem tooth loss. Chen et al. [24] found no significant changes in mandibular shape between the ages of 9 and 11 years old, and some significant changes between 11 and 15 years old which coincided with the onset of the pubertal growth spurt.

Different populations may show differences in mandibular size or shape based on evolutionary or genetic factors [25]. There may also be trends linked to environmental factors such as diet [26] which influences functional demands and the shape or size of the mandible over different time periods [27, 28]. These differences must be considered when trying to predict the shape of the mandible for any one individual from any one time period as there can be morphological plasticity in the shape of the mandible through time [29].

In context with other research into the shape of the mandible, Lavelle [30] looked at the mandibular shape of a sample of 90 female patients aged 12–15 years, with 30 class I, 30 class II and 30 class III by using LCRs. He used the technique of medial axis transformation of the mandibular outline form originally described by de Souza and Houghton [31]. The results showed consistency between the overall mandibular shape outline in all three groups. The medial axis lengths were all shorter (average 9%) in class II cases and all longer (average 11%) in class III cases [30]. These findings suggest while the size and position of the mandible may vary based on skeletal pattern, there may be less variation in the outline of the shape of the mandible.

Šidlauskas et al. [32] investigated the genetic and environmental impact on mandibular morphology using twin based studies in both monozygotic and dizygotic twins who had completed mandibular growth. The results showed that the saddle angle (NSBa) showed high genetic determination as well as the sagittal relationship of the mandible to the cranial base. In addition to this, the gonial angle was also under high heritability but linear measurements such as mandibular body length, ramus width and ramus height were more likely due to environmental factors or non-additive genetic factors [32]. Manfredi et al. [33] similarly found that mandibular structure seemed more genetically determined than mandibular size.

Other researchers have assessed different landmarks and measurements and related them to the shape of the mandible. Neha et al. [34] looked at the size and dimensions of the ST and whether there was any correlation with the size of the mandible or maxilla. Their results showed a correlation between the S length and area with both mandibular ramus height and mandibular body length and that the ratio between these measurements and S area was found to be nearly constant [34]. The area of S could potentially be used with the length of the anterior cranial base (SN) in order to more accurately predict the length of the mandibular body.

As the mandibular condyle articulates with the glenoid fossae of the temporal bone of the skull at the TMJ, attention has been paid to the shape of the glenoid fossae and the shape of the mandible. Kantomaa [35] investigated the correlation between the shape of the glenoid fossae and the morphology of the mandible and found that the inclination of the glenoid fossae in relation to the SN line correlated strongly with the configuration of the mandible. A vertically orientated articulating surface of the glenoid fossae seemed to direct condylar growth more vertically than a more horizontal articulating surface and therefore the inclination of the glenoid fossae may be a useful predictor for assessing skeletal divergency or vertical position of the MdP.

Halozenetis et al. [36] investigated the shape of the mandible from Art to Gn and used a Fourier analysis to analyse the shape of the mandible at circumpubertal timepoints. The results showed that the angles GoGn-SN, Frankfort horizontal-mandibular line (FH-ML) and Pal-GoGn were moderately to highly correlated to the shape of the mandible at all time points. These findings are of interesting relevance, as both SN and Pal (ANS-PNS) were measured in our study design. When used in conjunction with Go-Gn, which can be used as a method to determine the MdP, it is unsurprising these angles had a high correlation to the shape of the mandible around the circumpubertal period.

One of the key challenges is determining the size, position and shape of the inferior border of the mandible and delineating the MdP. This is commonly assessed by cephalometric analysis using the Maxillary Mandibular Plane Angle or Frankfort Mandibular Planes Angle. Where this angle is increased, this suggests that the individual is ‘high angle or hyperdivergent’ and where the angle is decreased, the individual is classified ‘low angle or hypodivergent’. Oh et al. [37] used mandibular landmarks to predict adult facial vertical types as described above. They found that Go, Me and Art were the best discriminating landmarks for predicting vertical facial pattern. As Go-Me can be used to determine MdP, it is expected that those would be useful discriminating landmarks for predicting vertical facial pattern. It is surprising that Art was one of the best discriminating landmarks for predicting adult facial vertical type and therefore this could have some application in conjunction with the inclination of the glenoid fossae [35] in predicting skeletal divergency.

Ayoub et al. [38] looked at whether the angle of the mandible could be used to differentiate between males and females (sexual dimorphism) in a Lebanese population with normocclusion and balanced facial proportions. They measured the mandibular angle using the ramal plane (Pr) and three different methods of determining the MdP: Down’s analysis using Go-Me [16], Steiner’s analysis using Go-Gn [17] and the Sassouni analysis using Go-Me [44]. The results showed that the female mandibular angles were slightly smaller than the males but that the mandibular angle could not be used as a differentiator for gender in a Lebanese population [38]. These findings suggest that neither gender nor the size of the angle of the mandible is particularly useful characteristics which may help predict the shape of the mandible in a Lebanese population. Alarcón et al. [39] also found that sex-specific mandibular traits behave differently across vertical facial patterns. In contrast to these findings, Schmittbuhl et al. [40] found that 84.1% of males and 81.2% of females presented significant sexual dimorphism of the shape of the mandible after size normalisation, while Franklin et al. [41] found the subadult mandible is not dimorphic. These differences in findings are likely down to differences in methodology between the studies, and therefore, while males tend to have larger sized mandibles than females, it is unclear from the current available evidence whether there are distinct differences in the shape of the mandible between males and females at different ages.

It is important to state at this point how accurate two-dimensional cephalometric measurements translate to anthropometric measurements of the face in reality. This gives an idea to how useful this prediction would be for anthropometric facial reconstruction. Budai et al. [42] investigated the relationship between anthropometric and cephalometric measurements of the face of human adult Caucasian participants. The results showed 97.4% of facial surface measurements and 96.7% of cephalometric measurements were considered normal. The cephalometric upper face, nose and upper lip heights did not differ significantly from the anthropometric counterparts but the cephalometric face height, lower face height and lower alveolar height were all lower in the cephalometric measurements than the anthropometric measurements. This suggests that two-dimensional cephalometric mandibular prediction may underestimate the vertical position or size of the mandible relative to the anthropometric measurements, although these differences are likely to be small.

## Conclusions

The novel method described in this study can potentially be used in conjunction with a lateral cephalometric radiograph to provide information that would be of use in predicting the two-dimensional shape of the human mandible. This may be extremely useful in forensic anthropology and archaeological reconstruction, where the mandible is often the bone missing when crania are discovered.

The most reliable parameter found to be predictable was mandibular length in the sagittal plane, particularly the skeletal landmarks gonion and pogonion. This method will allow this step in the prediction of mandibular shape to be used by forensic scientists and archaeologists in the reconstruction of missing mandibles.

However, the applicability of the results will need further investigation, predominantly due to the difficulty in determining the vertical position of the mandibular plane and the mix of different skeletal classifications in the subject radiographs assessed. It is, however, a promising first step and future research evaluating class I skeletal patterns only may yield further useful information. In forensic science and anthropology, and archaeological reconstruction, where the mandible may be missing, estimating the vertical position of the mandibular plane remains a challenge and it would not be possible to accurately reconstruct the vertical position of the inferior border of the mandible using the method described in this study alone, although other researchers have suggested other possible landmarks and correlations which may be useful in predicting skeletal divergency.

Future research should focus on determining the vertical position and inclination of the inferior mandibular border from other existing cephalometric landmarks or planes. This could then supplement the method described in this study and make the findings more applicable to predicting the two-dimensional shape of the mandible. Landmarks that were not assessed in this study but may be useful in predicting the two-dimensional shape of the mandible include sella, the inclination of the glenoid fossae of the TMJ and articulare. It would also be interesting to analyse a wider sample of untreated skeletal class I controls that have not been referred into secondary care for orthodontic or orthognathic treatment to see if a similar accuracy in estimating gonion is found in a larger sample. The results may then be more generalisable and representative of a more normal population.

## Data Availability

The data that support the findings of this study are available from Kingston Hospital but restrictions apply to the availability of these data, which were used under licence for the current study, and so are not publicly available. Data are however available from the authors upon reasonable request and with permission of Kingston Hospital.

## Abbreviations

ANS: Anterior nasal spine
Art: Articulare
CI: Confidence interval
FH-ML: Frankfort horizontal-mandibular line
FMPA: Frankfort mandibular plane angle
Gn: Gnathion
Go: Gonion
GoGn-SN: GonionGnathion-sella-nasion
Go-Me: Gonion-menton
ICC: Intraclass correlation coefficient
IQR: Interquartile range
LCR: Lateral cephalometric radiograph
MdP: Mandibular plane
Me: Menton
mm: Millimetres
MMPA: Maxillary mandibular plane angle
NHS: National Health Service
NSBa: Nasion-sella-basion
O: Point of origin
OG: Mandibular base plane
ON: Palatal plane
OP: Occlusal plane
OS’: Anterior cranial base plane
PACS: Picture archiving and communication system
PNS: Posterior nasal spine
Pog: Pogonion
Pr: Ramal plane
S: Sella
SN: Sella-nasion
S^p^: Most posterior point of the sella turcica
ST: Sella turcica
TMJ: Temporomandibular joint
